# Intelligence

**Published:** 1829-05

**Authors:** 


### INTELLIGENCE.

### MONTHLY REPORT OF PREVALENT DISEASES.

During the whole course of our professional experience, we do not remember to have found disease in general so frequent as it has been during the period which has elapsed since our last report. In this remark we are not singular: the same observation has been made by those practitioners with whom we are acquainted, who have the best opportunities of forming correct opinions upon the subject, both from their private and public practice. Fortunately, however, the severity of the prevailing maladies has not kept pace with their frequency. Many cases of catarrhal affection have not proved very obedient to the remedies prescribed; but, although obstinate in their duration, they have at last subsided without inducing any permanently bad effects. Pneumonic inflammation has been common, and severe in some instances, and also somewhat intractable; but still, upon the whole, we believe the ratio of fatality, in reference to the frequency of disease, has not been great.

Children have continued to suffer, during the last month, from measles, hooping-cough, and scarlet fever. Although we by no means coincide in the opinion that medical treatment, judiciously directed, is but of little service in hooping cough, we are convinced that Dr. DEWEES rates much too highly the efficacy of our art when he states, "that its duration may as certainly be shortened as the march of fever;" unless, indeed, the disease is characterised by much less determined features on the other side of the Atlantic than it is in this country. Some time ago we frequently administered the prussic acid, which has been so highly extolled, for the relief of this disease. We have now given up its use altogether in hooping-cough, as we have not found any benefit from its employment, and we do not hold ourselves justified in administering so very powerful a remedy, unless the advantages arising from it are decided.

---

*Royal College of Surgeons.*—Mr. GUTHRIE commenced his Surgical Lectures on Tuesday last, the 28th April. He will successively treat on the following subjects: Lecture 1st. On the natural and artificial means of suppressing Hemorrhage. 2d. On the nature and theory of Aneurism. 3d. On wounded Arteries, and the inapplication of the theory of aneurism to their treatment. 4th. On Inflammation of the Veins. Four lectures will succeed on Injuries of the Head.

---

*Jacksonian Prize.*—The Jacksonian Prize has been awarded by the College of Surgeons to Mr. GEORGE ROGERSON for the best dissertation on Inflammation of the Membranes.

---

*Regulations of the Apothecaries.*—Much anxiety has been created amongst many of our junior brethren, by an assertion which has lately been made in the Lancet, that certificates of lectures attended during the term of apprenticeship would *not* be received at Apothecaries' hall. The following letter, with which we have been favored by Mr. WATSON, will of course relieve any such groundless apprehensions:

*To the Editors of the London Medical and Physical Journal.*

Gentlemen,—I beg leave to request you will afford me a small space in your very useful Journal, for the purpose of contradicting, in the most unqualified manner, a misstatement contained in the Lancet of the 18th instant. My attention was directed to this *mis-leading* article by a medical student, who applied to me in my official capacity this morning, to ascertain whether there was any foundation for the great alarm these paragraphs had occasioned him.

The misstatement I allude to is as follows: "The whole scheme of examination at Rhubarb hall is well adapted to the powers of the examiners, seeing that it is made to depend on the inspection of certificates, and the verification of dates; *but the grand test of qualification is that whereby it is ascertained that no part of the candidate's extra-official knowledge has been acquired during the five years which must be exclusively devoted to the services of the shop.*

No degree of knowledge, no amount of professional acquisitions, will avail the applicant for a licence at Rhubarb hall. On the knowledge or professional acquisition of the candidate, the worshipful examiners do not, for the best of reasons, undertake to deliver any opinion: what they require is, the production of certificates showing that the candidate has attended certain courses of lectures: *but if the date of these certificates happen to fall within the five years required to be consumed in the drudgery of a shop, this is a fatal objection to the candidate's admissibility,* and he is rejected as incompetent to discharge the duties of a medical practitioner. In vain may the candidate urge that he is ready to undergo the most searching examination: it is useless, say the worshipful tradesmen, to urge your pretensions; *we can only examine your certificates, and your certificates are dated at a time when your whole attention should have been devoted to the services of the shop.*"

I shall make no observations on the vulgar and disrespectful terms in which the editor of the Lancet speaks of the Court of Examiners, in several passages of the article from which these extracts are copied ; because I presume not to enter upon the labour of attempting to reform either his language or his manners. The *only* motive I now have for taking any notice of what has appeared in the Lancet, arises from my desire to set at ease the minds of the great majority of students who are at the present time attending lectures.

If I were to designate the statements contained in the Lancet by the epithets which deservedly belong to them, I should be obliged to make use of terms which are not tolerated among gentlemen; but, as I wish not to depart from that propriety of language which our profession were accustomed to in their communications, before the Lancet introduced its peculiar vocabulary, I will only give to them the less offensive name of misrepresentations.

The Court of Examiners, I assert, have never, from the very first day on which the Court was formed to the present hour, refused to admit any candidate to an examination because he had attended any part, or the whole, of the required lectures during the period of his five years' apprenticeship: nor have they ever had, at any time, any intention of making a regulation to that effect. The Court of Examiners are fully sensible of the great benefit which medical students derive from attendance on lectures during their apprenticeship ; and they have on this account given their countenance and support to the medical schools which have within a short period been established in Manchester, Liverpool, Birmingham, Bath, Bristol, Leeds, and Sheffield.

The records of the Court contain ample testimony that more than two thirds of the persons examined did attend available courses of lectures during their apprenticeship ; and the book in which the names of the rejected persons, and the reasons for their rejection, are registered, proves that no candidate has been refused a certificate on any other ground than a deficiency of knowledge and of professional acquirements, on which the editor of the Lancet says the "examiners do not undertake to deliver any opinion."

Allow me, gentlemen, to request you will be good enough to give an early insertion to this letter, as I am anxious to dispel the alarm which the misstatement of the Lancet has occasioned to medical students.

I have the honour to be, gentlemen,

Your very obedient servant,

JOHN WATSON,

*Secretary to the Court of Examiners.*

*Apothecaries' Hall; April 18th,* 1829.

*Webb-street Medical School.*

*Medals to the meritorious Students of* Dr. HOPKINS's *Class.*—Medals and prizes impartially bestowed upon deserving students are the most judicious incentives to industry: they often succeed in calling into useful exercise that talent which otherwise would have lain dormant; and no doubt the starting point in many a course of successful scientific pursuit has been the rivalry of a contest for such youthful honours. It is, therefore, with much approbation, and with the hope that other teachers will more frequently follow the example, that we notice the adjudication of Dr. Hopkins's annual medals, which lately took place in the Webb street Theatre.

This subject of this year's Essay was "The Symptoms of Pregnancy from the earliest Stage to the Period of Quickening; with a Physiological Explanation of the Mental and Physical Changes produced by the impregnated Uterus upon the System of the Mother."

Seven gentlemen submitted papers for examination, all of which were very meritorious productions; but those of Mr. J. MORLEY and Mr. J. COOPER were declared, by an impartial jury composed of several of Dr. Hopkins's distinguished professional brethren, to be decidedly the best: accordingly, to the former candidate was allotted the gold medal, and to the latter the silver one.

The Essays themselves were indeed very admirable: they proved not only that their authors had profited by the able lectures of their teacher, but that, by the most diligent research, they had made themselves acquainted with the accumulated experience of almost all ages and countries. Dr. COPELAND, who was one of the judges, declared himself perfectly surprised that such Essays should be produced by students; they would not, he said, have disgraced veterans in the profession.

In order to prove to the class at large that the medals were not undeservedly bestowed, the test of a public examination was resorted to; each of the prize men being called upon first to read his Essay, then to explain and defend its reasonings and conclusions, under a rigorous *viva voce* examination; afterwards answering such questions on the obstetric science generally as Dr. H. thought fit to address to them. These examinations were so ably supported as to prove that the respondents were familiar not only with the particular subject of their Essays, but also with the science generally.

The medals are very superb; they are executed by Rundle, of Ludgate hill, in the first style of art. The gold one is very massive, containing four ounces of metal.

---

We are authorised to state that the vacancies in the medical departments of the Russian army and navy have been filled up, and that foreign medical officers will not be received hereafter into the imperial service.

---

*Foundling Hospital.*—Mr. G. J. PERRY was elected surgeon to the Foundling Hospital, on Wednesday, April 1st, in the room of Mr. EARLE, who resigned the office. The candidates were Mr. PERRY, Mr. SKEY, and Mr. TUSON.—Mr. Earle was honoured, on his resignation, by a very flattering vote of thanks from the committee, for the most zealous execution of the duties of his office for the term of sixteen years.

---

A vacancy has occurred in the appointment of one of the physicians to the General Dispensary, Aldersgate street, by the resignation of Dr. WOODFORDE.

### PRIZE QUESTIONS.

*Société de Médecine de Bruxelles.*—" To describe the state of the science of Medicine at the end of the eighteenth century, and to point out the progress that has been made in it, in a practical point of view, until the present time." Prize, a gold medal value 400 francs. Memoirs to be written in Latin, French, or Dutch; and to be transmitted, free of expense, before the 1st of August, 1829, to Dr. J. UYTTERHOEVEN, secretary to the Society, No. 1235, Rue Vinket, Brussels.—The members of the Society are not permitted to write for this prize.

---

*Société de Médecine de Metz.*—1. " Are there any cases in which death may occur without any appreciable organic lesion? 2. This question being answered in the affirmative, to prove, by cases or experiment, the possibility of such a kind of death. 3. To explain the *modus agendi* of the cause or causes of death in such cases."

The Society is anxious that, in the solution of this question, the writers of Essays should endeavour to show the inferences that may be derived from their arguments in a medico-legal point of view.

Prize, a gold medal value 300 francs. Memoirs to be addressed to M. CHAUMAS, secretary to the Society, before the 1st September, 1829.

---

*Société de Médecine de Paris.*—A prize of 300 francs, given to the Society by Dr. DENEUX, resident member, for the best essay upon the following subject: " What are the diseases produced by pregnancy? what diseases does pregnancy cure? and what diseases are arrested in their progress by pregnancy?"

Memoirs to be addressed, before the 31st October, 1829, to M. NACQUART, secretary general of the Society, Rue St. Avoie, No. 39.

---

### LITERARY NOTICE.

*Compendium of Anatomy.*—We recommend to the attention of anatomical students a very correct and well-arranged little work, which has recently been published by Mr. TUSON, under the title of " A Pocket Compendium of Anatomy." Although Mr. Tuson has compressed into a small space the description of the anatomical structure of the human body, he has given a very perspicuous and instructive summary of the subject.

### METEOROLOGICAL JOURNAL,

By Messrs. HARRIS and Co. Mathematical Instrument Makers, 50, High Holborn.

| March | Rain gauge. | Moon. | Thermom. 9 A.M. | Thermom. MAX. | Thermom. M N. | Barometer. 9 A.M. | Barometer. 10P.M. | De Luc's Hygrom. 9A.M. | De Luc's Hygrom. 10P.M. | Winds. 9 A.M. | Winds. 10P.M. | Atmospheric Variations. 9 a.m. | Atmospheric Variations. 2 p.m. | Atmospheric Variations. 10 p.m. |
|---|---|---|---|---|---|---|---|---|---|---|---|---|---|---|
| 20 | | ○ | 57 | 59 | 47 | 29.44 | 29.73 | 55 | 55 | SWva | S W | Fine | Fine | Cloudy |
| 21 | | | 55 | 60 | 38 | .95 | .93 | 55 | 55 | SSE | E | — | — | Fine |
| 22 | | | 50 | 51 | 42 | .71 | .62 | 55 | 53 | SE | E | — | — | — |
| 23 | | | 46 | 49 | 38 | .64 | .77 | 53 | 50 | E | E var. | — | — | — |
| 24 | | | 39 | 42 | 28 | .77 | .74 | 50 | 53 | E | E | — | — | — |
| 25 | | | 35 | 46 | 35 | .80 | .80 | 53 | 52 | ENE | E | — | — | — |
| 26 | | | 39 | 45 | 39 | .83 | .83 | 52 | 52 | E | E | — | — | — |
| 27 | | | 45 | 51 | 33 | .79 | .69 | 52 | 52 | SE | SE | — | — | — |
| 28 | | ☾ | 40 | 46 | 37 | .53 | .34 | 50 | 50 | E | E | — | — | — |
| 29 | | | 41 | 46 | 40 | .22 | .15 | 54 | 60 | E | E | Rain | Cloudy | Show'ry |
| 30 | .11 | | 44 | 47 | 40 | .10 | .10 | 64 | 63 | ENE | ENE | Foggy | Cloudy | Cloudy |
| 31 | | | 42 | 44 | 35 | .10 | .14 | 63 | 60 | ENE | NE | Cloudy | Cloudy | Rain |
| April | | | | | | | | | | | | | | |
| 1 | .09 | | 40 | 45 | 32 | .26 | .32 | 60 | 56 | NE | WNW | Fine | Fine | Cloudy |
| 2 | | | 36 | 41 | 32 | .35 | .44 | 56 | 57 | W | W | Snow | Show'ry | Cloudy |
| 3 | .03 | ● | 42 | 48 | 38 | .54 | .60 | 57 | 57 | NNW | WNW | Fine | Fine | Fine |
| 4 | | | 48 | 49 | 40 | .54 | .38 | 57 | 58 | WSW | SSW | — | — | Rain |
| 5 | | | 46 | 54 | 44 | .25 | .16 | 58 | 59 | SW | SW | — | Show'ry | Cloudy |
| 6 | | | 51 | 59 | 42 | .10 | .04 | 60 | 60 | SSW | SW | Cloudy | Rain | Cloudy |
| 7 | .28 | | 43 | 54 | 40 | .03 | .14 | 60 | 61 | SW | WSW | Fog | Rain | Fine |
| 8 | | | 44 | 50 | 38 | .26 | .27 | 61 | 63 | SSW | SSW | Show'ry | Show'ry | Fine |
| 9 | .63 | | 42 | 50 | 40 | .06 | .16 | 63 | 63 | SSE | WSW | Rain | Show'ry | Cloudy |
| 10 | | | 48 | 52 | 40 | .30 | .40 | 63 | 58 | SW | WSW | Cloudy | Fine | Fine |
| 11 | | ☽ | 50 | 53 | 44 | .46 | .35 | 58 | 58 | WSW | S | Fine | Fine | Rain |
| 12 | .34 | | 50 | 57 | 47 | .24 | .15 | 58 | 59 | SW | SW | Rain | Fine | Show'ry |
| 13 | | | 54 | 54 | 43 | .04 | .04 | 59 | 62 | SW | SW | Cloudy | Rain | Cloudy |
| 14 | .50 | | 53 | 57 | 47 | .14 | 28.91 | 62 | 56 | SSW | S | Cloudy | Rain | Fine |
| 15 | .35 | | 51 | 54 | 45 | 28.94 | 29.17 | 56 | 60 | SWva | SW | Cloudy | Cloudy | Fine |
| 16 | | | 47 | 53 | 40 | 29.11 | .21 | 60 | 62 | SW | NW | Cloudy | Rain | Cloudy |
| 17 | .50 | | 51 | 55 | 45 | .51 | .60 | 62 | 61 | WSW | WSW | Fine | Show'ry | Fine |
| 18 | .10 | | 53 | 56 | 43 | .65 | .65 | 61 | 61 | WSW | WSW | Fine | Show'ry | Cloudy |
| 19 | | ○ | 47 | 57 | 41 | .60 | .59 | 61 | 57 | SW | WSW | Fine | Show'ry | Fine |

The quantity of Rain fallen in the month of March, was 16-100ths of an inch.

